# Assessing the economic burden of Alzheimer’s disease patients first diagnosed by specialists

**DOI:** 10.1186/s12877-016-0303-5

**Published:** 2016-07-11

**Authors:** Noam Y. Kirson, Urvi Desai, Ljubica Ristovska, Alice Kate G. Cummings, Howard G. Birnbaum, Wenyu Ye, J. Scott Andrews, Daniel Ball, Kristin Kahle-Wrobleski

**Affiliations:** Analysis Group, Inc., 111 Huntington Ave, 14th floor, Boston, MA 02199 USA; Eli Lilly and Company, Lilly Corporate Center, Indianapolis, IN 46285 USA

**Keywords:** Alzheimer’s disease, Specialist, Primary care, Medicare, Cost

## Abstract

**Background:**

It is not known if there is a differential impact on Alzheimer’s disease (AD) diagnosis and outcomes if/when patients are diagnosed with cognitive decline by specialists versus non-specialists. This study examined the cost trajectories of Medicare beneficiaries initially diagnosed by specialists compared to similar patients who received their diagnosis in primary care settings.

**Methods:**

Patients with ≥2 claims for AD were selected from de-identified administrative claims data for US Medicare beneficiaries (5 % random sample). The earliest observed diagnosis of cognitive decline served as the index date. Patients were required to have continuous Medicare coverage for ≥12 months pre-index (baseline) and ≥12 months following the first AD diagnosis, allowing for up to 3 years from index to AD diagnosis. Time from index date to AD diagnosis was compared between those diagnosed by specialists (i.e., neurologist, psychiatrist, or geriatrician) versus non-specialists using Kaplan-Meier analyses with log-rank tests. Patient demographics, Charlson Comorbidity Index (CCI) during baseline, and annual all-cause medical costs (reimbursed by Medicare) in baseline and follow-up periods were compared across propensity-score matched cohorts.

**Results:**

Patients first diagnosed with cognitive decline by specialists (*n* = 2593) were younger (78.8 versus 80.8 years old), more likely to be male (40 % versus 34 %), and had higher CCI scores and higher medical costs at baseline than those diagnosed by non-specialists (*n* = 13,961). However, patients diagnosed by specialists had a significantly shorter time to AD diagnosis, both before and after matching (mean [after matching]: 3.5 versus 4.6 months, *p* < 0.0001). In addition, patients diagnosed by specialists had significantly lower average total all-cause medical costs in the first 12 months after their index date, a finding that persisted after matching ($19,824 versus $25,863, *p* < 0.0001). Total per-patient annual medical costs were similar for the two groups starting in the second year post-index.

**Conclusions:**

Before and after matching, patients diagnosed by a specialist had a shorter time to AD diagnosis and incurred lower costs in the year following the initial cognitive decline diagnosis. Differences in costs converged during subsequent years. This suggests that seeking care from specialists may yield more timely diagnosis, appropriate care and reduced costs among those with cognitive decline.

## Background

Alzheimer’s disease (AD) is a progressive neurodegenerative disease and the most common form of dementia in those over the age of 65 [1]. Approximately 5.3 million Americans have AD and this number is projected to be 13.8 million by 2050 [2].

Total payments in 2015 for healthcare, long-term care, and hospice care for Americans aged 65 years and older with dementia were estimated to be $226 billion [2]. In addition, it is estimated that more than 15.7 million family members and other unpaid caregivers provided an estimated 17.9 billion hours of care to people with AD and other dementias, a contribution valued at $217.7 billion in 2014 [2]. Further research suggests that the healthcare system may be paying for additional direct medical costs incurred by spouses and household members of persons with AD [3, 4].

Given that AD is an age-related disease and that most dementia cases are for persons over the age of 65, Medicare has been, and will remain, the main funding source for medical treatment of patients with AD. However, recent legislation [5] acknowledges that the current healthcare system is not designed to address the complexities of care management for AD patients. Furthermore, dementia-related quality measures are not yet an integral part of the current healthcare system [6] and there is a need to identify ways to improve the diagnosis and outcomes for AD patients.

An accurate and timely initial dementia diagnosis may help patients and caregivers better plan for managing the underlying disease and may lower costs to healthcare systems [7, 8]. However, detection and diagnosis of dementia can be challenging because of the complex presentation of symptoms, many of which overlap with manifestations of other conditions [9]. Literature has shown that this is particularly problematic in the primary care setting. For example, in a study by Borson et al. [10], primary care physicians correctly identified only 41 % of cognitively impaired patients. A meta-analysis of detection, diagnosis, and documentation rates in the existing primary care environment suggests highly variable detection rates for the presence of cognitive impairment. Diagnosis rates are generally higher in more severe stages, but documentation rates for dementia and cognitive impairment remain low [11].

Though the constraints of primary care practice related to dementia diagnosis have been examined, it is not known if there is a differential impact on AD diagnosis and outcomes if/when specialists are included in care. For example, given the complexities of diagnosis and management, diagnosis of AD by specialists, who have additional training and expertise in this area, may be preferable. However, this approach could increase costs of care and create additional hurdles in the healthcare system due to the low number of available specialists. For example, Dall et al. [12] estimate a 19 % shortfall of neurologists by 2025. It is therefore important to better understand the links between care setting at the time of dementia diagnosis and subsequent outcomes (e.g., healthcare resource use and costs).

The current study looks to inform the literature by examining the cost trajectories of Medicare beneficiaries initially diagnosed with cognitive decline by specialists compared to similar Medicare recipients who received their diagnosis in primary care settings. If differences exist, the type of difference may be informative for discussions related to establishing quality metrics and/or best practices.

## Methods

### Data

This study used data from the Medicare Standard Analytical files containing de-identified administrative claims for a 5 % random sample of fee-for-service Medicare beneficiaries (~3-million). Prior to 2009, these data did not have precise dates of service available. Having a precise date of service was necessary to characterize key outcomes of our study (e.g. time to AD diagnosis). This analysis, therefore, used the most recent years available (2009 through 2012) to identify patients diagnosed with cognitive decline. The data include patient demographics (age and gender), enrollment history, medical diagnoses received and associated dates of service, place of service (e.g., hospitalizations, physician office visits, emergency department (ED) visits), provider specialty, and paid amounts. Prescription drug claims were not available.

### Sample selection

**Fig. 1:**
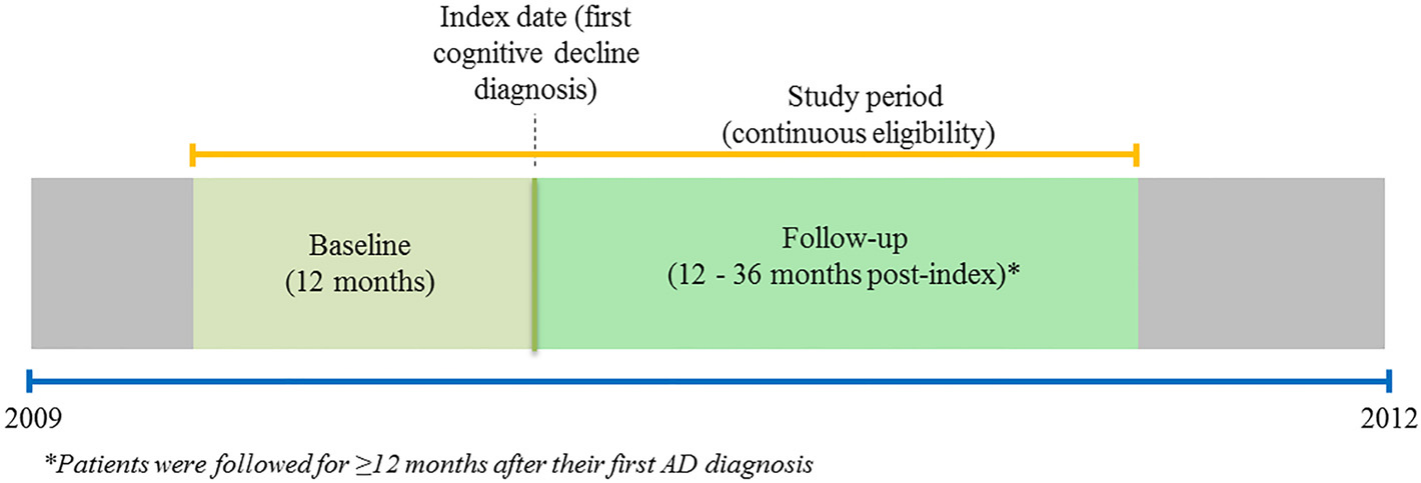
Study time periods

Medicare beneficiaries with at least two distinct claims with a diagnosis code for Alzheimer’s disease (International Classification of Diseases, Ninth Revision, Clinical Modification [ICD-9-CM] code 331.0), with the first such claim occurring in the period 2009 through 2012, were identified as AD patients. The AD patients identified in this same period (2009 through 2012) whose first claim for cognitive impairment (“index date”; ICD-9-CM: 290.x, 291.1, 291.2, 292.82–83, 294.x, 331.x, 780.93) was before or at the date of AD diagnosis were also included in the analysis. Continuous Medicare enrollment was required during the 12 months prior to the index date (baseline period), the period between the index date and the first AD diagnosis, and the 12 months following and including the first AD diagnosis, resulting in a follow-up period of at least 12 months and up to 36 months after the index date (Fig. 1). Patients were then stratified into two cohorts depending on whether they were first diagnosed as having cognitive impairment (i.e., on their index date) by a specialist or not. A diagnosis was considered to be made by a specialist if the corresponding claim listed the physician specialty as a neurologist, psychiatrist, or geriatrician.

### Propensity score matching

AD patients diagnosed with cognitive impairment by a specialist were matched one-to-one by greedy matching algorithm to patients diagnosed in a primary care setting using propensity scores [13], length of follow-up, and medical costs in the baseline period (±1/10 of the average baseline costs among patients diagnosed by a specialist). Propensity scores (i.e., likelihood of being diagnosed by a specialist) were estimated using a logistic regression model that accounted for differences in baseline characteristics across the two cohorts, including patient-level demographics (age, gender, and year of index date), Charlson Comorbidity Index (CCI) [14], hyperlipidemia, and depression.

### Outcomes

Time to AD diagnosis, defined as time from the index date (i.e., earliest observed indication of cognitive decline) to AD diagnosis, was compared between the patients diagnosed by a specialist and those diagnosed by non-specialists. All-cause medical costs from the payer perspective (i.e., payments by Medicare to providers in 2012 US dollars) were evaluated in each year of follow-up among matched pairs of patients with continuous Medicare eligibility for the duration of the entire year being evaluated. Costs were further categorized by place of service in order to identify the main sources of differences in medical costs. Categories of service included: hospital inpatient, hospital outpatient, physician office, home health, ED, skilled nursing facilities, durable medical equipment, and hospice. Since prescription drug claims were not available, drug costs were not included in the analysis.

### Statistical analyses

For categorical variables in baseline and follow-up, statistical significance of differences between patients treated by a specialist and controls was assessed using chi-squared tests prior to matching and McNemar tests after matching. For continuous variables, statistical significance was evaluated using Wilcoxon rank-sum tests prior to matching and Wilcoxon signed-rank tests after matching. Kaplan-Meier survival analysis was used for time to AD diagnosis comparison between the patients diagnosed by a specialist and those diagnosed by non-specialists. Statistical analyses were performed using SAS version 9.3 (SAS Institute Inc., Cary, NC). A *p*-value of < .05 was considered as statistically significant.

## Results

The selection criteria resulted in a final analytic sample of 16,554 patients, of whom 2593 (15.7 %) were diagnosed by a specialist and 13,961 (84.3 %) were diagnosed by non-specialists.

### Time to AD diagnosis

**Fig. 2:**
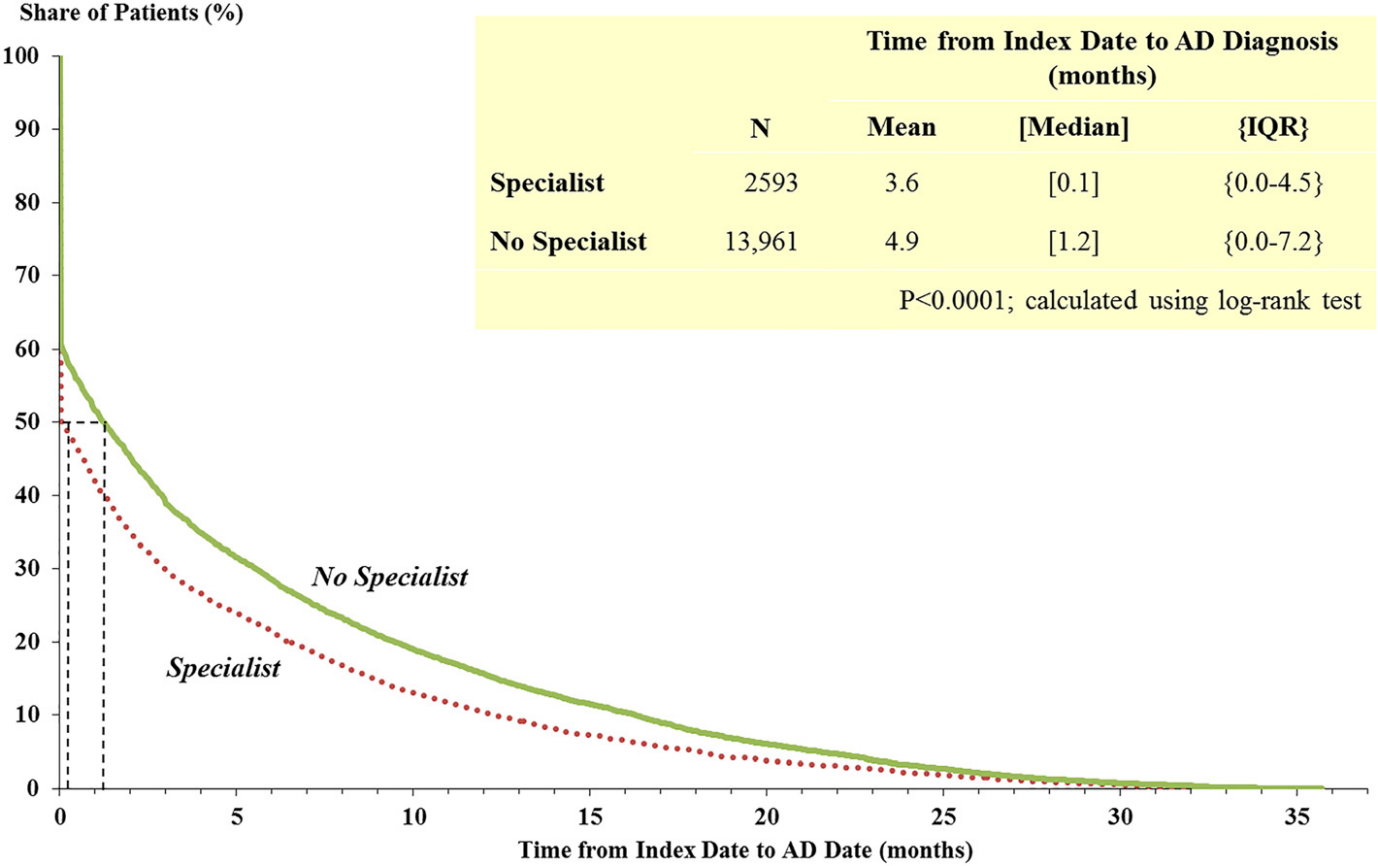
Time from index date to AD diagnosis date. NOTE: patients diagnosed by specialists versus no specialists (before matching)

Patients who received their first cognitive impairment diagnosis by a specialist had a statistically significantly shorter time to AD diagnosis (median 0.1, mean 3.6 months) compared to patients diagnosed by non-specialists (median 1.2, mean 4.9 months) (Fig. 2). Results were similar after propensity score matching (Table 1). Of note, 50 % of those diagnosed by specialists received an AD diagnosis on their index date, compared to 40 % of those diagnosed by non-specialists.

**Table 1:** Patient characteristics during the 12 months prior to the index date

Selected Characteristics	Before Matching			After Matching		
	Specialist (*n* = 2593)	No Specialist (*n* = 13,961)	*p*-Value*	Specialist (*n* = 2503)	No Specialist (*n* = 2503)	*p*-Value**
Age on index date, mean (SD)	78.8 (8.7)	80.8 (8.2)	<0.0001	79.1 (8.3)	79.3 (8.1)	0.3593
% male	40 %	34 %	<0.0001	40 %	39 %	0.4781
Charlson Comorbidity Index, mean (SD)	2.2 (2.2)	1.9 (2.1)	<0.0001	2.1 (2.1)	2.1 (2.1)	0.5307
Comorbidities, %						
Chronic obstructive pulmonary disease	18 %	17 %	0.8061	17 %	18 %	0.2465
Depression	20 %	18 %	0.0890	19 %	20 %	0.4097
Diabetes	31 %	29 %	0.1159	30 %	33 %	0.0123
Epilepsy	4 %	2 %	<0.0001	3 %	2 %	0.0080
Hyperlipidemia	62 %	52 %	<0.0001	61 %	63 %	0.1431
Hypertension	78 %	76 %	0.0270	77 %	80 %	0.0059
Ischemic heart disease	37 %	31 %	<0.0001	36 %	35 %	0.1537
Other cardiovascular conditions	60 %	55 %	<0.0001	59 %	58 %	0.4371
Stroke/cerebrovascular disease	28 %	20 %	<0.0001	27 %	20 %	<0.0001
Urinary tract infection	22 %	21 %	0.7036	21 %	23 %	0.0579
Time to AD diagnosis, mean (SD)	3.6 (6.2)	4.9 (7.2)	<0.0001	3.5 (6.2)	4.6 (6.8)	<0.0001
Months of follow-up, mean (SD)	29.8 (9.8)	31.2 (10.2)	<0.0001	29.9 (9.8)	30.1 (10.0)	0.0105
Total healthcare costs, mean (SD)^a^	$15,058 ($26,139)	$13,339 ($24,356)	<0.0001	$12,497 ($18,789)	$12,503 ($18,797)	0.5597

*Calculated using Wilcoxon-rank sum tests for continuous variables and chi-square tests for categorical variables

**Calculated using Wilcoxon signed-rank tests for continuous variables and McNemar’s tests for categorical variables

^a^Dollar values were inflated to 2012 US dollars using the medical care component of the Consumer Price Index

Note: Patients were one-to-one matched based on patients’ age, gender, year of index date, Charlson Comorbidity Index, hyperlipidemia, and depression by propensity scores greedy method

### Baseline characteristics

Prior to matching, AD patients first diagnosed with cognitive impairment by a specialist were younger (78.8 versus 80.8 years, *p* < 0.0001) and more likely to be male (40 % versus 34 %, *p* < 0.0001) compared to those diagnosed by non-specialists. Additionally, patients diagnosed by specialists had significantly higher rates of comorbidities such as hyperlipidemia (62 % versus 52 %, *p* < 0.0001), stroke/cerebrovascular disease (28 % versus 20 %, *p* < 0.0001), and cardiovascular conditions including hypertension and ischemic heart disease (Table 1). Furthermore, those diagnosed by specialists had higher total all-cause medical costs at baseline ($15,058 versus $13,339, *p* < 0.0001) and had, on average, 1.4 fewer months of follow-up time available compared to those diagnosed by non-specialists (Table 1).

The matching process resulted in identification of 2503 matched pairs of AD patients who were initially diagnosed with cognitive impairment by a specialist and AD patients diagnosed by non-specialists. The matched cohorts had similar demographic characteristics and baseline healthcare costs. However, those diagnosed by specialists had higher rates of epilepsy and stroke, but lower rates of diabetes and hypertension at baseline compared with their matched counterparts (Table 1).

### Costs during the follow-up period

**Fig. 3:**
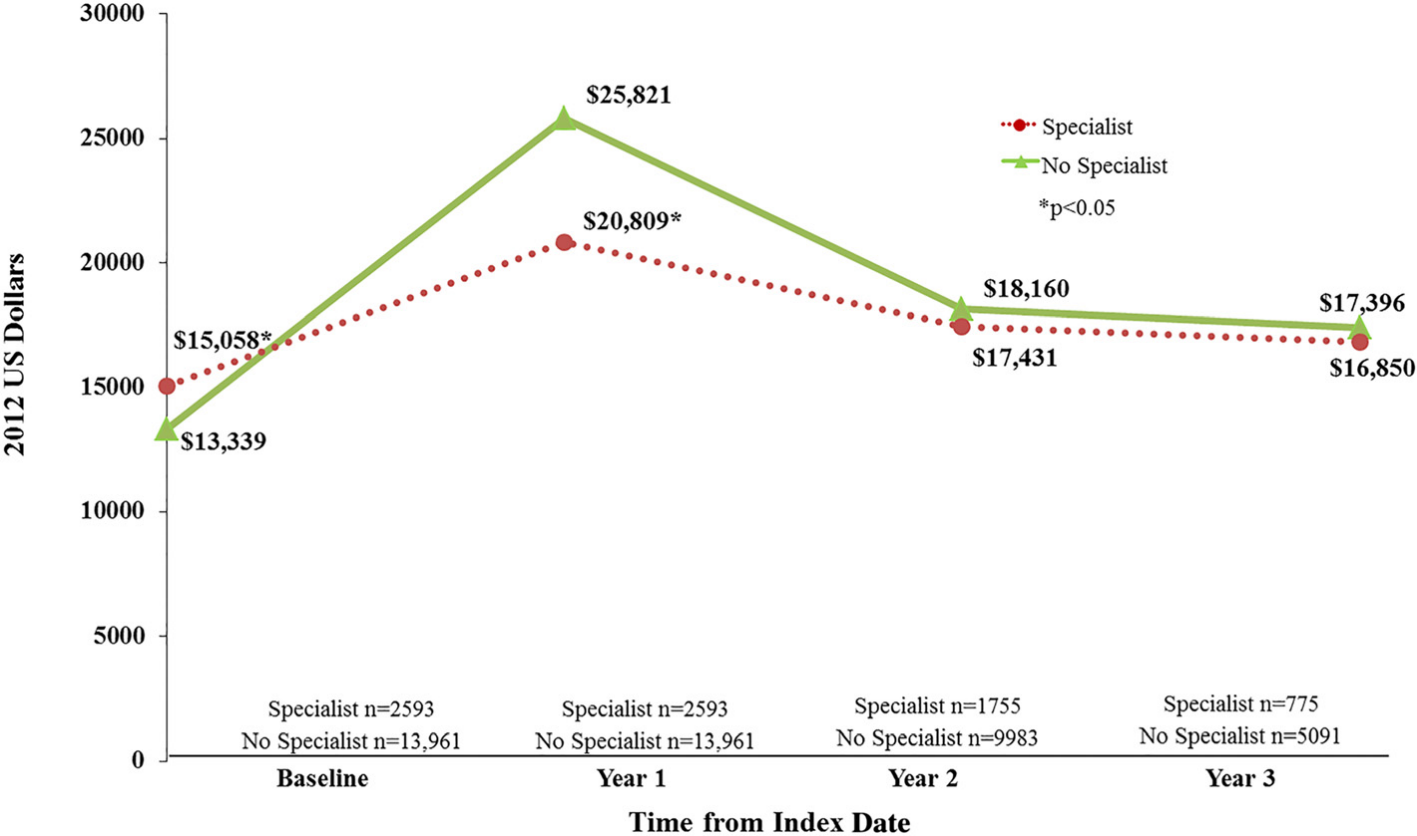
Average total all-cause costs in follow-up period. NOTE: patients diagnosed by specialists versus no specialists (before matching)

**Fig. 4:**
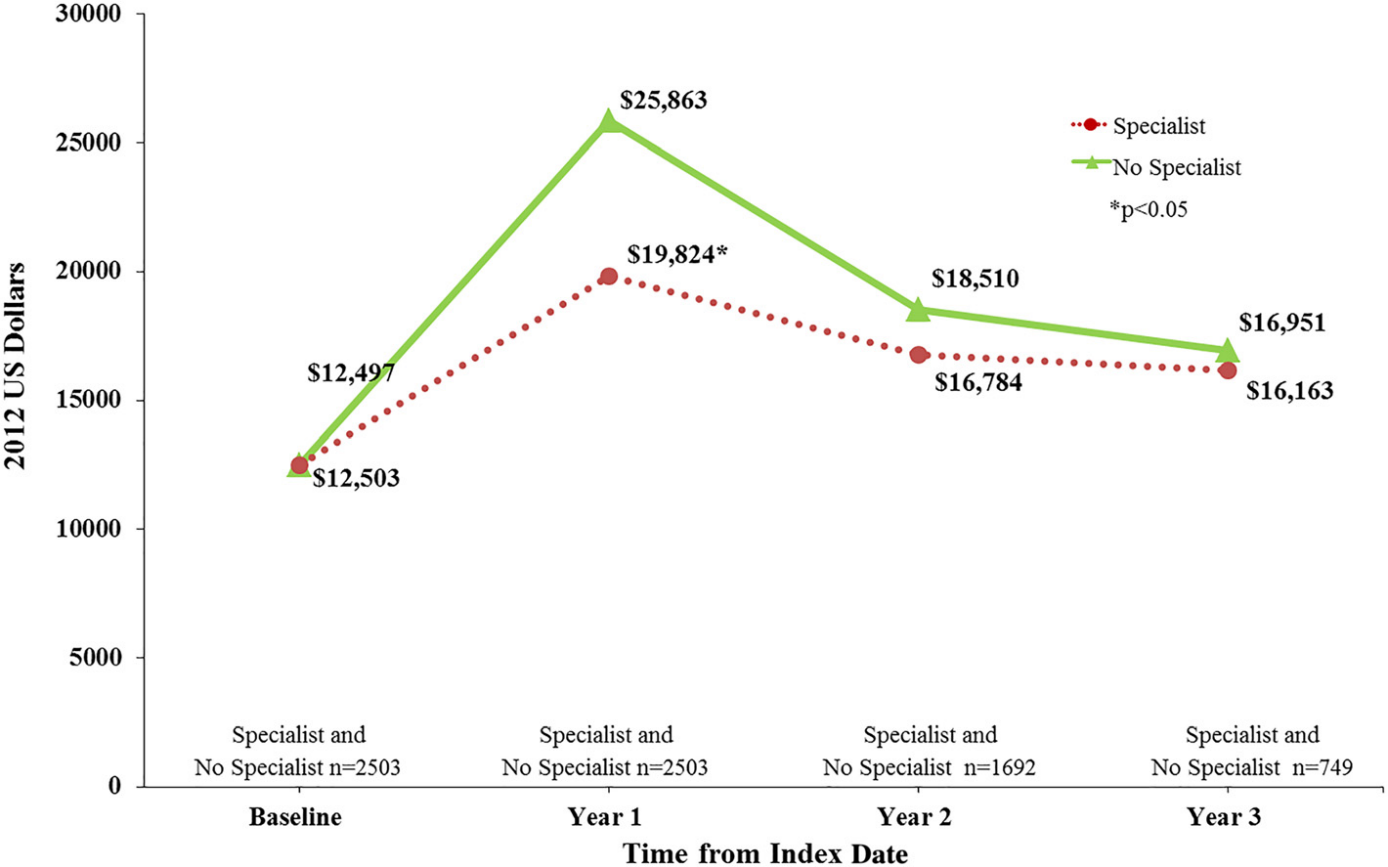
Average total all-cause costs in follow-up period. NOTE: patients diagnosed by specialists versus no specialists (after matching)

Before matching, patients first diagnosed with cognitive impairment by a specialist had significantly lower average total all-cause medical costs in the first 12 months after their index date ($20,809 versus $25,821, *p* < 0.0001; Fig. 3). This cost differential persisted even after accounting for underlying differences using propensity score matching ($19,824 versus $25,863, *p* < 0.0001; Fig. 4). In the second and third years of follow-up, the two cohorts had statistically similar costs, both before and after matching (Figs. 3 and 4).

**Fig. 5:**
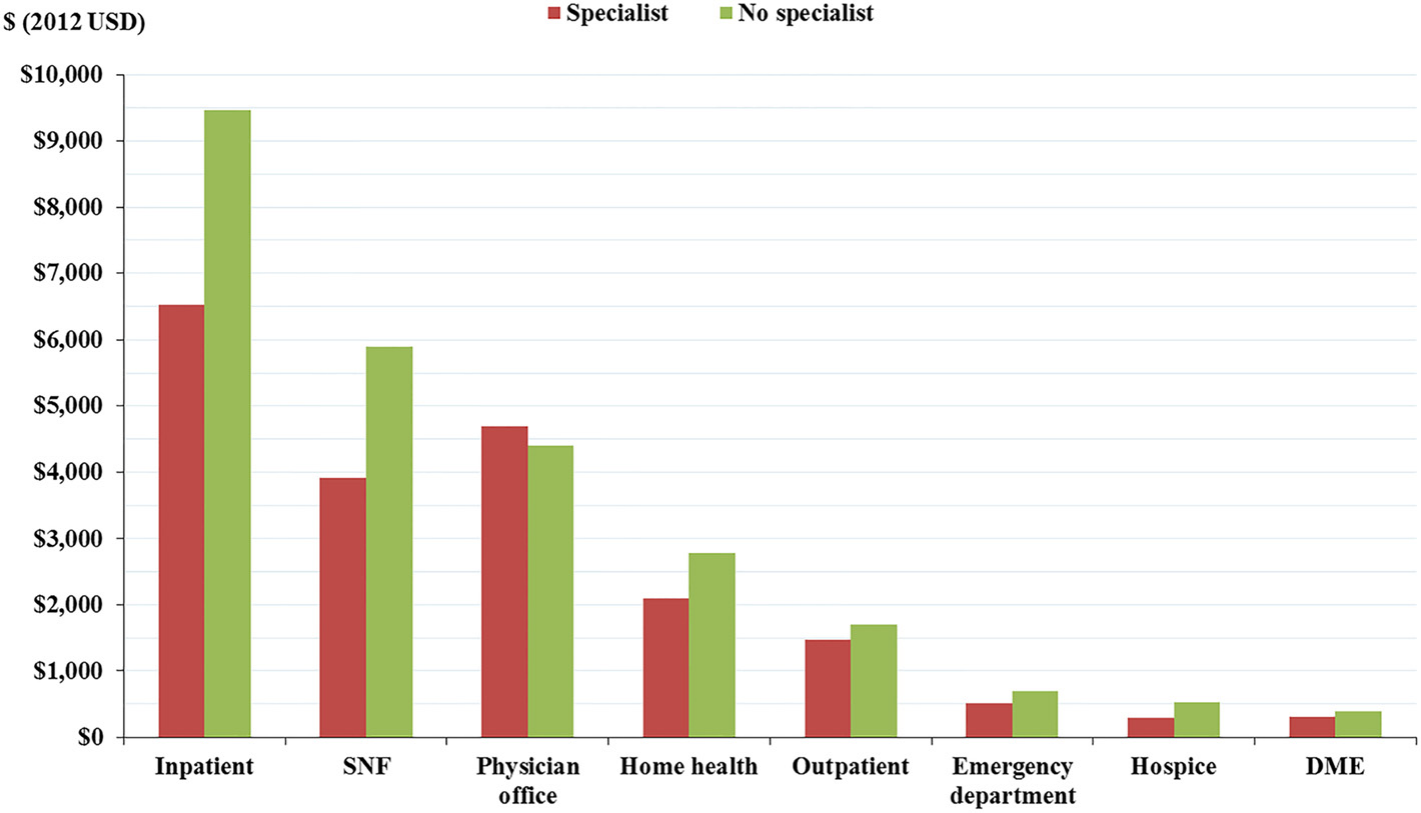
Components of costs in Year 1 of follow-up period. NOTE: patients diagnosed by specialists versus no specialists (after matching). Abbreviations DME = durable medical equipment; SNF = skilled nursing facilities; USD = United States dollars. Note: Differences across cohorts are statistically significant at *p*<0.05 for all places of service. Inpatient stays include stays originating from the emergency department

In terms of costs by place of service during the first year of the follow-up period, AD patients first diagnosed with cognitive impairment by a specialist had significantly lower costs, on average, in every place of service compared to the matched control population, with the exception of physician office visits (Fig. 5). The difference in inpatient costs (−$2940, *p* < 0.0001) comprised 48.7 % of the difference in total medical costs among the two cohorts, followed by the difference in skilled nursing facility costs, which accounted for 32.9 % (−$1986, *p* < 0.0001) of the overall all-cause medical cost differential.

## Discussion

Consulting a specialist upon first indications of cognitive decline appears to be associated with a modestly shorter time until diagnosis of AD. The shorter time to diagnosis is primarily because patients seen by a specialist are more likely to receive an AD diagnosis on the index date as opposed to a general diagnosis of cognitive impairment prior to a more definitive diagnosis of AD. This may be attributable to the fact that specialists have more expertise in identifying dementia symptoms and therefore may be more comfortable making a diagnosis.

An alternative explanation could be related to the differential rates of various comorbidities across the two cohorts. For example, after matching, patients first diagnosed by specialists had significantly higher rates of epilepsy and stroke/cerebrovascular disease, but patients who were not first diagnosed by specialists had significantly higher rates of diabetes and hypertension. Conditions like epilepsy and stroke are likely already managed by a specialist, so patients and their caregivers may be more likely to self-refer to a specialist if additional or worsening cognitive symptoms occur, thus resulting in a faster diagnosis. Primary care physicians may also more readily refer patients with a history of a major neurological disorder to a specialist. On the other hand, primary care providers may view symptoms of cognitive decline in patients with diabetes and hypertension as part of those chronic diseases and thus feel that an additional diagnosis is not warranted or may take more time to confirm that the cognitive deficit is significant enough to merit a separate evaluation, focusing instead on more controlled management of diabetes and hypertension. However, recent work by Barnes et al. [15] suggests that a history of type II diabetes should be part of the primary care algorithm for identifying patients at high risk for cognitive impairment. Broader dissemination of these types of criteria and increased awareness by primary care providers about risk factors for AD and other dementias may help shorten the diagnostic process. It is also worth considering that different types of patients may present to primary care physicians versus specialists.

Prior to matching, patients diagnosed by specialists had higher CCI scores and costs at baseline, despite being younger. This would suggest that more complex patients are seen by specialists. The expectation might therefore be for higher costs to accrue after diagnosis as well. On the contrary, the present analyses suggest that patients diagnosed by a specialist have lower costs in the year following initial diagnosis. This pattern holds even after matching on baseline characteristics including demographics and all-cause medical costs.

In keeping with the National Alzheimer’s Project Act goal of earlier diagnosis of AD, these results suggest that seeking timely care from specialists may result in a quicker diagnosis and reduced overall medical costs among those with cognitive decline. These trends should be monitored in the coming years as more patients make use of benefits provided under the Affordable Care Act, such as the Medicare Annual Wellness Visit [16], which includes detection of any cognitive impairment among eligible individuals (i.e., those who have had Medicare Part B for longer than 12 months). The results of this study suggest potential advantages of a specialist performing the exam for certain individuals at risk for AD. However, given the complexity of the disease and its presentation, the best use of resources may be for simple detection in the primary care setting followed by timely referral to a specialist. An expected shortage in the availability of specialists makes it unlikely that more timely receipt of an accurate diagnosis will be achieved without initiatives that also focus on improving the diagnosis of AD by primary care physicians. This may become even more important in the future, as evidence is accumulating that the biggest potential for delaying progression and/or modifying the disease trajectory appears to be when intervening earlier in the disease continuum [17].

Although the present study provides some preliminary data on possible benefits of specialist care for diagnosis, limitations of claims data prohibit definitive statements about which care setting is optimal. These analyses rely on the accuracy of the ICD-9 coding of diagnoses in administrative claims data, which do not necessarily reflect confirmed clinical diagnoses and lack information to assess the severity of illness. A recent report noted that fewer than 50 % of individuals with AD reported being told of their diagnosis [2]. To the extent a similar pattern of under-reporting of AD diagnoses exists in billing claims, this analysis does not characterize the full cohort of AD patients in Medicare. It is unclear to what extent the omission of these patients from our analysis may impact our results. If the omitted AD patients are earlier in the spectrum of disease, they may be less likely to be seen by specialists and also cost less, thereby lowering the average cost of the ‘No specialist’ cohort. In addition, having detail on the severity of AD (e.g. Mild, Moderate or Severe) would provide useful insights as to whether the lower Year 1 costs among those treated by specialists hold across all severity levels or whether the aggregate difference is driven by a particular severity cohort. Regardless of the completeness of the AD cohort and the lack of specificity on severity, these results do describe the cost trends stratified by type of treating physician at the time of diagnosis of cognitive decline among individuals eventually coded as having AD in Medicare claims. Further research using data sources that allow more complete and accurate identification of AD patients, including by dementia severity, would be a valuable addition to knowledge in this area.

The severity of symptoms at the time of diagnosis is not known and may impact how well a patient is managed in primary versus specialty care. The level and quality of coordination between the primary care provider and specialist is also not known. For example, lower costs of care in patients seen by specialists may be due to efforts of proactive primary care providers, caregivers, and patients who reach out more quickly to specialists and take a more active care management and coordination role. In addition, costs do not reflect prescription drug use, medical services covered by other payers (e.g., Medicare/Medicaid dual eligibility), or over-the-counter medications or informal care.

Additionally, while the propensity score matching analysis controlled for observable differences across patient cohorts, it cannot account for unobserved heterogeneity. Furthermore, in addition to a more timely diagnosis, treatment by a specialist may also result in a more detailed, tailored treatment plan that allows for better patient management and thus lowers costs. However, the present study did not evaluate whether or not patients continued to receive specialist care following their initial diagnosis.

This study excluded individuals diagnosed prior to 2009 in order to utilize precise dates of service that were not available in the data prior to that year. Restricting the cohort to a more recent sample may somewhat limit the generalizability of these findings. However, for more than 50 % of the sample, the first observed cognitive diagnosis was for AD. In addition, for those with a first diagnosis of cognitive impairment, the median time from that diagnosis to AD diagnosis was less than one month. These factors, combined with a mean duration of follow-up similar between the two cohorts, suggest that limiting the sample to 2009 through 2012 would not have a substantial impact on our findings.

It is likely that AD will continue to exert a growing and significant burden on health and social care systems. Better understanding how best to adapt the healthcare system to this impending epidemic may help improve patient and system-level outcomes. Additional research as to the most beneficial roles of specialists and primary care providers may help expand on the current analyses to help policy-makers reach better informed decisions on if, how, and when to implement broader screening, detection, and diagnostic protocols for Medicare beneficiaries. Future research should consider the application of these analyses in the broader dementia population given the challenges of differential diagnosis. Also, additional research is warranted to understand the impact of continued specialist care among patients with cognitive decline.

## Conclusions

In conclusion, these analyses demonstrated that both before and after matching, patients not diagnosed by a specialist incurred higher costs in the year immediately following the initial cognitive decline diagnosis. In Years 2 and 3, costs in both cohorts remained higher than baseline though the difference between them narrowed and was not statistically significant at those time points. These results suggest that seeking care from specialists may result in more timely diagnosis and reduced costs among those with cognitive decline.

## Abbreviations

AD, Alzheimer’s disease; CCI, Charlson Comorbidity Index; ED, emergency department; ICD-9-CM, International Classification of Diseases, 9^th^ Revision, Clinical Modification
